# Red Palm Oil Attenuates Lead Acetate Induced Testicular Damage in Adult Male Sprague-Dawley Rats

**DOI:** 10.1155/2015/130261

**Published:** 2015-09-21

**Authors:** A. I. Jegede, U. Offor, O. O. Azu, O. Akinloye

**Affiliations:** ^1^Discipline Clinical Anatomy, School of Laboratory Medicine and Medical Sciences, Nelson R Mandela School of Medicine, University of KwaZulu-Natal, Durban 4013, South Africa; ^2^Anatomy Department, Faculty of Basic Medical Sciences, College of Health Sciences, Ladoke Akintola University of Technology, Ogbomoso, Oyo State 210001, Nigeria; ^3^Department of Medical Laboratory Science, Faculty of Basic Medical Sciences, College of Medicine, University of Lagos, Lagos 100001, Nigeria

## Abstract

To study the protective effect of Red Palm Oil (RPO) on testicular damage induced by administration of lead acetate on male Sprague-Dawley rats, 28 rats divided into four groups of 7 animals each were used. They were administered orally with RPO (1 mL and 2 mL) and lead acetate (i.p.) 6 mg/kg body weight/day, respectively. Treatment was conducted for 8 weeks, and 24 hrs after the last treatment the rats were sacrificed using cervical dislocation. Sperms collected from epididymis were used for seminal fluid analyses; while the testes sample was used for ROS and oxidative enzyme activities assessment. Statistical analysis was carried out using GraphPad Prism 5.02 statistical analysis package. Administration of lead acetate increased generation of reactive oxygen species (ROS) significantly (*p* < 0.05) as evidenced by the elevated value of H_2_O_2_ and LPO and decreased GSH level. Also there was reduced epididymal sperm count, poor grade of sperm motility, and lower percentage of normal sperm morphology significantly. Coadministration with RPO, however, has a protective effect against lead toxicity by decreasing H_2_O_2_ production, increased GSH level, and increased sperm qualities especially. This shows that RPO has a potential to attenuate the toxic effect of lead on testicular cells preventing possible resultant male infertility.

## 1. Introduction

Red palm oil (RPO) is produced from the fruit of the oil palm tree (*Elaeis guineensis*). It originated from tropical Africa and now spread to most part of the world. RPO is an important produce from oil palm in Southeast Asia, West Africa, and South America [1]. Throughout history RPO has served as the primary source of dietary fat. Its nutritional and healing properties have been recognized for generations. Until modern medicine arrived, RPO was the remedy of choice for several illnesses in many parts of Africa. RPO is regarded among many as essential in the diet for pregnant and nursing mothers in order to ascertain good health of the mother and child [2]. RPO is virtually regarded as powerhouse of nutrition containing by far more nutrients than any other dietary oil [3]. In addition to beta-carotene, alpha-carotene, and lycopene it contains at least 20 other carotenes along with vitamin E, vitamin K, CoQ10, squalene, phytosterols, flavonoids, phenolic acids, and glycolipids. RPO is one of the richest natural sources of vitamin E. In addition to ordinary vitamin E, it also contains the highest amount of a super potent form of vitamin E known as tocotrienol. There are four tocotrienols, which are all present in RPO. These tocotrienols have up to 60 times the antioxidant activity of ordinary vitamin E. The combination of vitamin E, tocotrienols, carotenes, and other antioxidants makes palm oil a super antioxidant food [3, 4]. RPO is the only vegetable oil with a balanced composition of saturated and unsaturated fatty acids in both processed and unprocessed forms [5]. It contains carotenoids, phosphatides, sterols, tocopherols, and trace metals [5], shown to be effective against oxidative stress* in vitro* and* in vivo* [6]. It has also been shown that RPO exerted effects on reproductive capacity by improving the efficiency of protein biosynthesis or utilization in such a way that was favorable to sex hormone function in rats fed with RPO [7, 8]. It is also likely that RPO provide vitamin A, which is known to play a part in reproduction through the synthesis of sex steroids [7], embryogenesis, and spermatogenesis [9].

Lead is a toxic heavy metal that can damage nervous connections (especially in young children) and cause blood and brain disorders. Lead poisoning typically results from ingestion of food or water contaminated with lead but may also occur after accidental ingestion of contaminated soil, dust, or lead based paint [10]. Long-term exposure to lead or its salts (especially soluble salts or the strong oxidant PbO_2_) can cause nephropathy and colic-like abdominal pains. Concentrations of blood lead >40 *μ*g/dL seem to be associated with a decrease in sperm count, sperm volume, sperm motility, and morphological alterations [11, 12]. Chronic, high-level exposure has shown to reduce fertility in male [13]. The effects of lead are the same whether it enters the body through breathing or swallowing. Lead is probably toxic to most organs and systems in the body. Chronic lead poisoning is commonly seen in young children from sucking lead paint or lead toys and in workers engaged in printing, paint, and petroleum industries [14]. Reproductive dysfunction by lead has distinct morphological and biochemical features such as disorganized epithelia, decreased sperm quality and altered sperm morphology, and low androgen levels [12, 15, 16]. In the animal model, lead has a primary toxic effect on the hypothalamic pituitary unit and a primary effect on the testes and acts at all levels of the reproductive axis [17].

Industrial lead exposure is rampant among people working in some industries such as paint and battery making companies. Leaded gasoline is still in use in many developing countries resulting in environmental lead pollution. The incidence of lead poison around the world remains unabated as recent cases are still reported in countries like Nigeria, China, and Japan [10–13, 16]. This calls for scientific research to find out substances especially natural products that could prevent or reduce lead toxicity in high risk population. This study was designed specifically to attenuate lead induced testicular damage.

## 2. Materials and Methods

### 2.1. Source of Chemicals

Red palm oil from Linkjon commercial processing oil mill, certified after quality indices were determined for moisture content, free fatty acid (FFA), acid value, and saponification value using standard methods by National Agency for Food and Drug Administration and Control (NAFDAC), was purchased from Sabo oil market in Ilesa, Osun State. Nigeria and all other chemicals were purchased from Sigma Chemicals, USA. The administered dose of lead acetate was selected according to our previous study [16].

### 2.2. Animals

Twenty-eight healthy adult male Sprague-Dawley rats (10 weeks old, 165 g average body weight) were housed in clean polypropylene cages and maintained in animal housing facility with constant 12 h/12 h dark and light cycle. All animal handling procedure was approved by the College Ethics Committee.

### 2.3. Experimental Protocol

The rats were divided into four groups (*n* = 7), labeled as groups I, II, III, and IV. Group I represents control and received water as* placebo*. Group II was administered (i.p.) with 6 mg/kgbw lead acetate only. Groups III and IV intraperitoneally received 6 mg/kgbw of lead acetate and 1 and 2 mL of RPO, respectively. Feed and water were made available* ad libitum.*

The administration lasted for 8 weeks. Lead acetate was administered intraperitoneally and palm oil was administered orally with the use of oral cannula. Twenty-four hours after the last treatment, the animals were sacrificed by cervical dislocation after which the testes and epididymis were removed for sperm assessment and all left testes were fixed in Bouin's fluid and epididymis in normal saline while all the right testes were stored at −80°C until analysis. Also the initial and final body weights of the rats were taken before the commencement of treatment and prior to sacrifice.

### 2.4. Seminal Fluid Analyses

The sperm count was done using new improved Neubauer's hemocytometer (Deep 1/10 mm, LABART, Germany). This procedure including evaluation of sperm motility and morphology was done as reported in our previous study [16].

### 2.5. Antioxidant Enzyme

Samples from testes were homogenized in 50 mM tris-HCL buffer (pH7.4) containing 1.15% potassium chloride and the homogenate cold (4°C) centrifuged at 10,000 ×g for 15 minutes. The supernatant was collected for the estimation of markers of oxidative stress; H_2_O_2_, LPO, and GSH levels were assayed as described by Adedara and Farombi, 2014 [18, 19].

### 2.6. Histological Procedure

Tissues were fixed in Bouin's fluid for routine H&E histological preparation and photomicrographs of the sections were taken at different magnifications.

### 2.7. Statistical Analysis

The results were statistically analyzed using the Prism 5 for Windows (version 5.02, GraphPad Software, Inc.). The Mean ± Standard Error of Means of the data was calculated. One-way ANOVA was used to check the significance differences within and between groups while Student's *t*-test was used to compare difference between means. The difference of means was considered significant at *p* < 0.05.

## 3. Results

*Mortality.* No mortality was recorded during the course of the experiment.

### 3.1. Body Weight

The body weight of animals in groups II and III was reduced compared to controls (*p* < 0.05) with 15.2% and 11.05% decrease in body weight, respectively, while the final body weight of group IV animals increased by 14.28% (Table 1).

### 3.2. Seminal Fluid Analyses

The sperm count of all the lead acetate groups was significantly reduced when compared to group I. Group II records a more overtly low sperm count which is significant compared to all other groups; however, there is no significant difference in sperm count between groups III and IV (Table 1). Sperm motility grading showed that there is a high cell death and reduced motility in group II compared to the control group and the groups that receive RPO treatment where 64% (group III) and 66% (group IV) rapid progressive motility were recorded against 26% recorded in group II (Table 1).

### 3.3. Testicular Oxidative Stress Status

**Figure 1:**
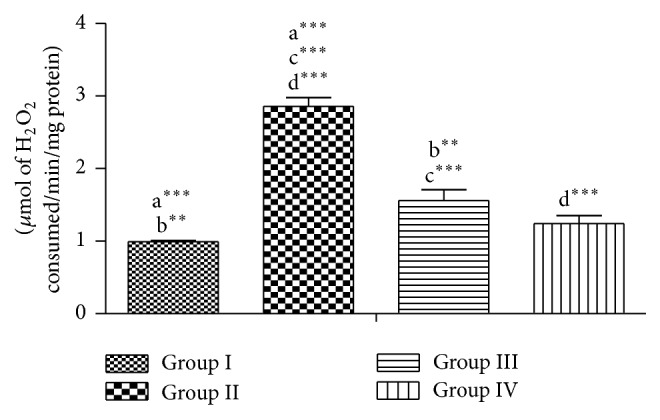
Effect of RPO on the H_2_O_2_ level in testes of rat treated with lead acetate. Each bar represents the mean ± SEM of 7 rats.

**Figure 2:**
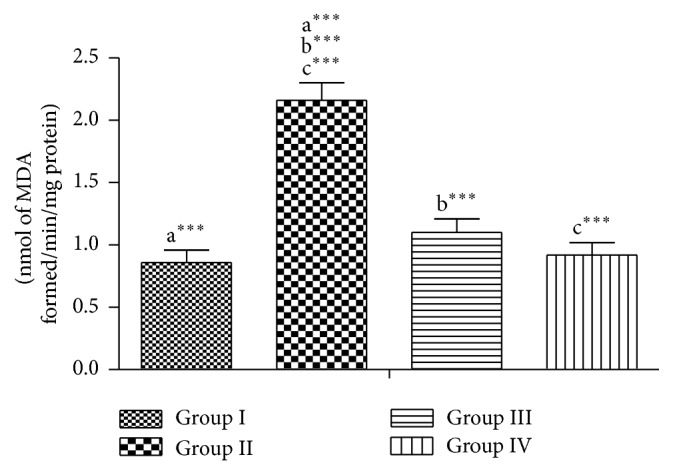
Effect of RPO on the LPO level in testes of rat treated with lead acetate. Each bar represents the mean ± SEM of 7 rats.

**Figure 3:**
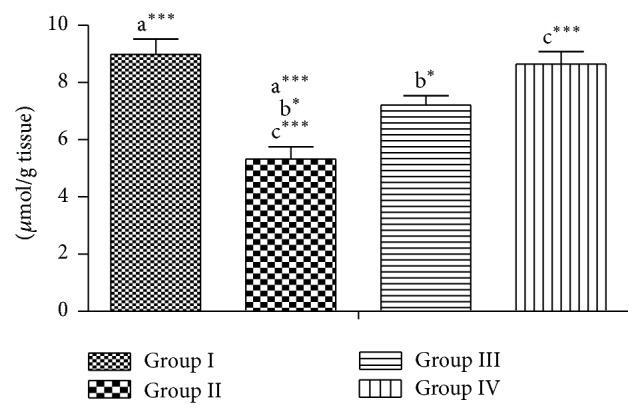
Effect of RPO on the GSH activity in testes of rat treated with lead acetate. Each bar represents the mean ± SEM of 7 rats.

Lead acetate significantly increased the levels of H_2_O_2_ and LPO and reduced the level of GSH (group II), while it is seen in groups III and IV that were coadministered with RPO that there are no significant changes in the enzymes activities when compared to group I, meaning that the testes were protected from lead which induces oxidative stress (Figures 1, 2, and 3).

### 3.4. Histology

**Figure 4:**
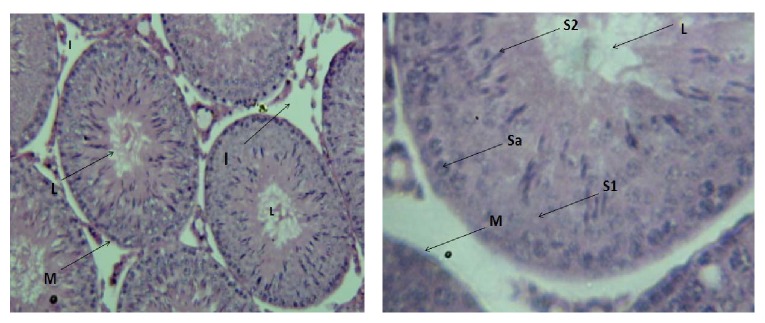
Cross section of the testis of rat in group I (H&E, ×100 and ×400, resp.). Lumen = L, basement membrane = M, interstitial space = I, spermatogonia = Sa, primary spermatocytes = S1, and spermatids = S2.

**Figure 5:**
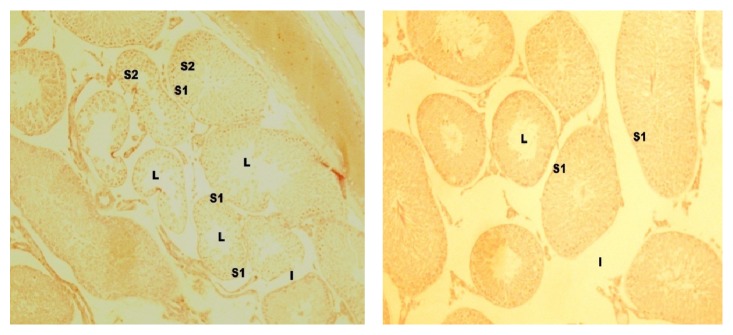
Cross section of the testis of rat in group II (H&E, ×40, ×100). S1 = spermatogonia cells, L = lumen, and I = interstitial spaces.

**Figure 6 fig6:**
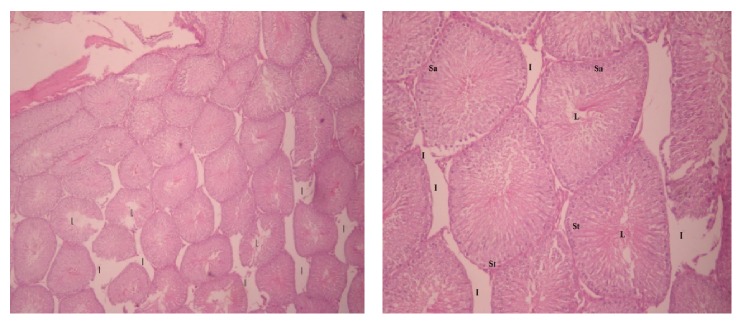
Cross section of group III (H&E, ×40, ×100, resp.). L = lumen, I = interstitial cells, Sa = spermatogonia, and St = primary spermatocyte.

**Figure 7:**
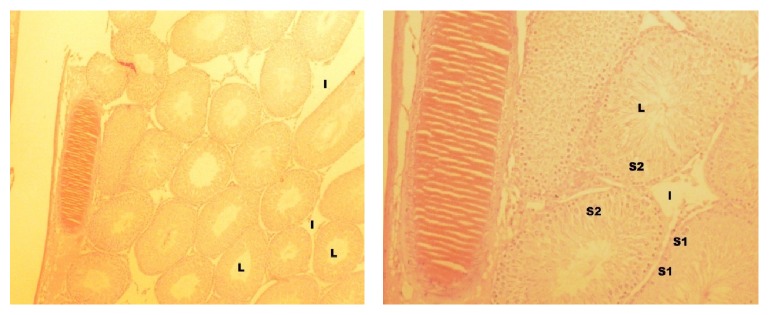
Cross section of group IV (H&E, ×40, ×100, resp.). L = lumen, I = interstitial space, and S2 = seminiferous tubule.

Figures 4–7 represent histological changes of testes in each group at two magnifications.

## 4. Discussion

Heavy metals are widely distributed in the environment. Environmental discharge of lead due to the use of petroleum products (especially leaded petrol), construction works, paint removal, demolition, vehicle batteries, and car repairs contribute to airborne lead pollution [20] and possibly introduce high concentrations of this potential reproductive toxicant into the environment, which may cause physiological, biological, and histological disorders [16, 21, 22]. Finding protection for these exposed individual becomes highly necessary. The present study highlights various deleterious impacts of lead acetate on the testicular parameters. We observed a statistically significant (*p* < 0.05) declined body weight (15.2%) in lead acetate only treated groups when compared to the control. Also weight loss of 11.05% was recorded in group treated with 1 mL of RPO against a weight gain of 14.28% in group treated with 2 mL RPO. RPO attenuated the weight loss in lead treated animals in doses dependent manner. The higher the doses of palm oil, the lesser the weight lost. Although the observed initial animal body weight range for the experiment is wide, this did not in any way significantly influenced the change observed. Whereas increased food consumption by the animals in RPO (higher dose group) is plausible, the overall effects of these changes are statistically factored in the design of the experiment.

The seminal fluid assessments (sperm count and sperm motility) were significantly decreased in the lead treated groups (II, III, and IV) compared with group I; this shows that lead toxicity causes oligospermia. Similar observations were recorded in experimental animal experimental models exposed to heavy metals [16, 21, 23, 24]. However, a slight increase was noticed in the groups treated with RPO in a dose dependent manner. This agrees with previous report that palm oil is a potential supplement to increased fertility [25]. The significant high level of dead sperm cells in group I shows that lead has adverse inhibitory effect on the postmeiotic cells, mainly pachytene spermatocyte and Leydig cell as seen in the complete erosion of the interstitial cell.

The percentage of the sperms with normal morphology in lead treated groups (groups II, III, and IV) was decreased with high prevalence of sperm head abnormality compared with control; this concurs with the earlier report which indicated that lead altered testis histology resulting in structural defects in sperms [26]. Also, the percentage of sperms with normal morphology was significantly increased in group II compared to group I; this is probably due to the protective effect of palm oil. The testicular histopathological evaluation of our result showed that lead produced germ cell apoptosis, spermatogenic arrest, testicular atrophy, and generalized degeneration of the interstitial space (Figures 5–7). It was seen that spermatogonia cells were the only features seen in group II; this implies that there is an arrest of the sperm development as no fully matured cell can be seen which implies that the process of spermatogenesis is affected by lead. This result agreed with the report of Al-Azemi et al. [27] where administration of Cd, another heavy metal, was shown to be spermatogenic state specific.

Reactive oxygen species (ROS) are highly reactive forms of oxygen, free radicals (FR), which are short-lived intermediate containing one or more unpaired electrons. They include an array that has superoxide anion (O^2−^), hydrogen peroxide (H_2_O_2_), hydroxyl radical (OH), nitric oxide (NO), hypochlorous acid (ClOH), peroxide (ROOH), and peroxynitrite (ONOO) [28]. These highly reactive species are generated in a number of conditions by cellular and acellular mechanism and have been implicated as an aetiological factor of a wide range of diseases. Studies have indicated that heavy metals act as catalysts in the oxidative reactions in biological reactions and that toxicity of these heavy metals may be due to oxidative tissue damage [21, 29]. The result of assay of level of H_2_O_2_ and LPO in this study shows a significant increase in the amount of generation of these free radicals in the lead acetate only treated group and a similar decrease in the RPO administered groups in a dose dependent manner. GSH is an intercellular antioxidant which is usually in high concentration within cell. In mice treated with heavy metals there were significant decreases in the levels of GSH and SOD in kidney and testicular tissues [21]; our result followed this similar pattern of low level of GSH in the lead acetate exposed groups. There was a high significant reduction in the GSH in group II compared to control group I (Figure 3). This marked low level of GSH is due to the excessive utilization of the antioxidant in scavenging for the high amount of free radicals produced by lead acetate. But this effect was reversed to a near normal state in group IV, where RPO was seen to alleviate the generation of free radicals by protecting the testicular tissue and hence a significant increase in the level of GSH. It can therefore be postulated that the protective action of RPO is similar to the role of intracellular GSH which is the most important protective mechanism for free radical scavenging and inhibitor of electrophilic xenobiotics attack on cellular macromolecules [30]. Preventative antioxidants, such as metal chelators and metal binding proteins, block the formation of new ROS, whereas scavenger antioxidants such as vitamins E and C, beta-carotene, and other antioxidants dietary supplements, glutathione and enzymes, remove ROS already generated by cellular oxidation. Dietary products such as vitamins C, E, and A are some of the excellent sources of antioxidant [31] and RPO is a virtual powerhouse of nutrition. It contains, by far, more nutrients than any other dietary oil. A new dimension to the view above from our study is to consider ranking RPO in the group of preventive antioxidants, since it appears to block the formation of ROS.

## 5. Conclusion

Our results suggest that RPO offers positive protection against lead acetate induced testicular injuries. However, further studies are warranted to elaborate on the definite mechanism of its antioxidative potential.

## Figures and Tables

**Table 1:** The Mean ± SEM of body weights of the rats and % body weight difference of the groups and seminal fluid analyses parameters.

	Group I	Group II	Group III	Group IV
Body weight (g)				
Initial weight	204.38 ± 5.74	121.80 ± 8.14	151.70 ± 3.82	118.90 ± 7.07
Final weight	210.50 ± 5.46	103.20 ± 7.55	134.94 ± 4.08	138.70 ± 6.78
Weight difference (%)	+6.12 (2.99)^abc^	−18.60 (15.2)^a^	−16.76 (11.05)^b^	+19.80 (14.28)^c^
Sperm count (×10^6^)	78.00 ± 7.8^abc^	15.70 ± 7.4^ade^	32.30 ± 3.3^bd^	34.90 ± 4.2^ce^
Motility grading (%)				
Rapid progressive	84.0 ± 1.0^abc^	26.0 ± 7.75^acd^	64.0 ± 5.10^bc^	66.0 ± 6.78^cd^
Slow progressive	8.0 ± 2.0^abc^	12.0 ± 2.0^a^	16.0 ± 4.0^b^	22.0 ± 3.7^c^
Nonprogressive	5.0 ± 0.0^a^	27.0 ± 5.8^abc^	11.0 ± 3.8^b^	7.0 ± 1.2^c^
Dead	5.0 ± 0.0^a^	32.0 ± 3.2^abc^	7.0 ± 5.8^b^	5.0 ± 0.0^c^
Morphology assessment (%)				
Normal	79.0 ± 2.92^abc^	40.0 ± 7.07^ad^	46.0 ± 2.45^b^	52.0 ± 3.74^cd^
Head defect	10.0 ± 2.24^abc^	42.0 ± 2.00^a^	40.0 ± 3.16^b^	38.0 ± 3.73^c^
Tail defect	6.0 ± 1.00	7.0 ± 1.23	6.0 ± 1.00	5.0 ± 0.0
Middle piece defect	5.0 ± 1.0^a^	11.0 ± 5.15^a^	6.0 ± 0.0	5.0 ± 1.0

Values with the same superscripts on the same row are statistically significant at *p* value <0.05 (*n* = 7).
